# Ethnic differences in prostate cancer

**DOI:** 10.1038/bjc.2011.273

**Published:** 2011-08-09

**Authors:** P Kheirandish, F Chinegwundoh

**Affiliations:** 1 Department of Urology, St Bartholomew's hospital, Barts and The London NHS Trust, West Smithfield, London EC1A 7BE, UK; 2 Department of Urology, Newham University Hospita lNHS Trust, Glen Road, London E13 8SL, UK.

**Keywords:** prostate cancer, ethnicity, incidence rate, aetiology

## Abstract

**Background:**

It is recognised that the risk of prostate cancer is higher in black men than in white men worldwide. Recent studies suggest that a number of genetic mutations in black men predispose them to this disease; hence, race as well as environmental factors such as diet and migration are thought to be the determining factors.

**Methods:**

This review compares data from the United States (US), which suggest that African-American men have a 60% higher risk for developing prostate cancer with poorer prognosis in comparison with their white counterparts, with similar studies carried out in the United Kingdom (UK) and also in African and Caribbean countries.

**Conclusions:**

Studies from the United States and the United Kingdom came to significantly different conclusions, and this has implications for policy development, awareness raising among black men in each country and clinical practice.

Over the last 30 years, prostate cancer rates in the United Kingdom (UK) have almost tripled, although much of the increase is due to increased detection through widespread use of the prostate-specific antigen (PSA) test (PSA is a 34 kDa glycoprotein enzyme produced by the columnar and ductal prostatic epithelial cells. It is used as a biomarker in diagnosis, staging and monitoring prostate cancer patients).

Worldwide, an estimated 913 000 men were diagnosed with prostate cancer in 2008, and more than two-thirds of cases were diagnosed in developed countries. The highest rates are in Australia/New Zealand, Western and Northern Europe and North America, largely because the practice of PSA testing and subsequent biopsy has become widespread in those regions (Gann, 1997; Ferlay *et al*, 2008).

As migration takes place and social demography changes, it is important to understand the disparity in incidence rate, management and prognosis among different ethnic populations in different countries.

It is difficult to compare the incidence rate of prostate cancer in black men in different countries because of the difference in detection pathways and data collection, but what is consistent in all published studies is the fact that black men are at a higher risk of developing prostate cancer and at younger age in comparison with their white counterparts (Parkin *et al*, 2003; Ferlay *et al*, 2008; Aus *et al*, 2005; Edwards *et al*, 2005).

In this manuscript, we explored the burden of prostate cancer among black men and compared it with their white counterparts in countries with a high proportion of black men. Our goal was not to provide a meta-analysis evaluation, nor conduct a comprehensive review of these studies. Our aim was to merely examine the available proof in an effort to better understand the burden of prostate cancer among black men of African ancestry and examine the suggested aetiologies behind the disparities in incidence rates and clinical course of this disease among black men in those countries.

## Materials and methods

To achieve the objective for this study, we conducted a literature review, summarising the body of evidence on prostate cancer incidence rate in countries with a high population of Black men of African ancestry. A systematic search of the computerised database, MEDLINE, and PUBMED was conducted for each country from the originating date of MEDLINE to July 2010 using the following keywords: black men, prostate cancer, prostate cancer risk, prostate cancer incidence and the name of relevant countries and continent such as United Kingdom, the United States (US), Caribbean and Africa.

The inclusion criteria were as follows:

- Original studies and review articles;
- Publication in English;
- Relevance to prostate cancer risk, incidence, prevalence or mortality; and
- Sufficient quality and quantity of evidence, based on study design and validity, for example, well-designed randomised/non-randomised study with good evidence to support study conclusion.

### Africa

It has been suggested that the disproportionate burden of prostate cancer among black men of West African ancestry follows the path of the Trans Atlantic Slave Trade (TAST) between 1450 and 1900 (Odedina *et al*, 2006).

The primary West African sources for the TAST were Nigeria, Benin, Ghana, Gambia and Senegal. Contrary to World Health Organisation's worldwide cancer data (GLOBOCAN, 2002) on prostate cancer incidence rates, a review study of all available articles on this subject from West African countries and Nigeria in prostate cancer incidence rate is in fact much higher than previously thought; for instance, it is 127 per 100 000 among Nigerian men compared with 19.3 per 100 000 reported in GLOBOCAN, 2002 (Osegbe, 1997; Ferlay *et al*, 2008).

Reports on incidence rates of prostate cancer in West African countries are scarce and mostly single hospital-based reviews focusing mainly on mortality and morbidity. However, in Nigeria, where multicentre studies are available (17 reports till 2006), the incidence rate of prostate cancer among Nigerian men (127 per 100 000) is closer to that of black men in the US (258 per 100 000; www.seer.cancer.gov; Odedina *et al*, 2009).

Bearing in mind that in West African countries access to health-care services and PSA testing is limited and no comprehensive population-based study is available yet, perhaps it can be concluded that the true incidence rate for prostate cancer in West African countries is higher than it is currently estimated. We believe that by having a cancer registry in West African countries, the true incidence rates of prostate cancer in those countries can be calculated more accurately. Where there is limited access to health-care providers, there is late presentation and higher PSA levels (Evans *et al*, 2010; Odedina *et al*, 2009).

### Caribbean

The study of clinical cases from Kingston, Jamaica (Glover *et al*, 1998) reported the highest incidence rate among black men in the world (304 per 100 000). Subsequent reviewers confirmed a high incidence in Jamaica, although not at the same level as in Glover (Hanchard *et al*, 2001; Blake *et al*, 2002).

### United Kingdom

The study of incidence rates and presenting features of prostate cancer among African-Caribbean, European and South Asian men in North East London was the first of its kind in the United Kingdom (Chinegwundoh *et al*, 2006). This study reports that the age-adjusted incidence rates were 647 per 100 000 for African Caribbeans, 213 for Europeans and 199 for South Asians. It showed a 3-fold relative risk for prostate cancer in black men compared with white men; a modest increase in presenting PSA levels for black men (mean PSA level of 42.4 for black men compared with 37.6 for European men); no significant difference in Gleason scores (Gleason score is a histopathological grading system and is used to help evaluate the prognosis of men with prostate cancer), but slightly worse clinical stage for black men (T4 and/or M1 (based on TNM staging system (2002), T4 represents tumours invading to adjacent structures and M1 represents distant metastasis), 26.4% for black men *vs* 23.0% for white men).

A subsequent and more comprehensive study on ethnicity and prostate cancer in the UK, PRostate Cancer in Ethnic SubgroupS (PROCESS), was published in 2008. This study found that black Caribbean men in the UK have the highest incidence rate of prostate cancer, followed by white men and South Asian men. It also showed that, although on average, African-Caribbean men are diagnosed 5 years earlier than white men, but all studied ethnic groups had equal access to diagnostic services. It also revealed that, with the exception of presenting PSA level, clinical presentation and management of prostate cancer were similar among all ethnicities.

The methods used in this study were innovative compared with previous studies. This was a cohort population-based retrospective study that compared (1) the incidence rate of prostate cancer among African Caribbean, European and South Asian ethnic groups; (2) the disparities in pathways to diagnosis; and (3) the clinical presentation and initial management of prostate cancer cases diagnosed in North Bristol, South West London, South East London and North East London for a period of 5 years between 1997 and 2001 (Ben-Shlomo *et al*, 2008; Evans *et al*, 2008; Metcalfe *et al*, 2008).

The PROCESS study group identified 2140 incident cases over the 5-year period between 1997 and 2001. The age-adjusted incidence rate for white men was 56.4 per 100 000. For black Caribbean men it was 173 per 100 000 and 139 per 100 000 for black African men. The average age-adjusted incidence rate for all black men in the UK was calculated at 166 per 100 000. The study concluded that the black men were three times more likely to be diagnosed with prostate cancer than white men, and this was more marked for younger men. African men had slightly lower relative risks than Caribbean men, but this was statistically insignificant (Ben-Shlomo *et al*, 2008).

The United States age-specific rates for black men in 1999 showed that the incidence rate for black men in the US was 283 per 100 000 and 172 per 100 000 for white men (http://www.seer.cancer.gov). Comparing these results show a greater relative risk of developing prostate cancer for black men in the UK than that seen in the US mainly because of the lower rate of prostate cancer in white men in the UK compared with their US counterparts. The greater use of PSA testing and inclusion of screen-detected cases can explain the higher incidence rate in both black and white men in United States.

The UK black men are largely first-generation migrants, but US black men have lived in the US for many generations. The higher incidence rate among US black men could be attributed to the effect of migration, although when comparing the incidence rates in different countries, the difference in detecting process in those countries cannot be ignored. Needless to say, further research is needed to confirm whether black men in Caribbean and Africa have similar, lower or higher rates than black men in the UK and in the United States so that we can have a clear idea as to the role of migration on prostate cancer risk.

Surveillance, Epidemiology and End Results (SEER) data from the United States indicate that black men are 2.4 times more likely to die of prostate cancer than white men of the same age (Edwards *et al*, 2005).

It is also suggested that black men in the US have worse access to healthcare generally and PSA testing in particular (Institute of Medicine, The National Academy Press, 2001)

Those alarming figures from United States prompted the PROCESS study group in the UK to investigate the pathway to prostate cancer diagnosis in black and white men and to explore whether race and socioeconomic status influenced prostate cancer diagnosis. The UK National Health Service has an ethos of health-care equality; hence it is possible that difference in diagnostic pathways followed by black and white men is avoided (Whitehead, 1994).

In the PROCESS study, men known to be alive at the time of the study were asked to complete a questionnaire including the 2001 census questions on ethnicity, with the next-of-kin being contacted if the man had died more than 6 months ago. The questionnaire also assessed demographic information, knowledge of prostate cancer and delays in presenting with symptoms.

Trained research nurses reviewed hospital records using a standard performa, extracting information on PSA measurements and histological investigations; investigations aimed at determining cancer stage and initial management strategies. An ecological measure of socioeconomic position was obtained by linking each man's home postcode to the corresponding 1998 electoral ward (http://www.edina.ac.uk/), and then to the Index of Multiple Deprivation score for the year 2000 (http://www.neighbourhood.statistics.gov.uk/). Six domains (namely income; employment; health deprivation and disability; education, skills and training; housing; and geographical access to services) determine the index score for an area, with higher scores indicating greater deprivation.

Measures of PSA level and tumour differentiation from the time of diagnosis and before commencing any treatment were identified.

A Delphi sub-study investigated the appropriate use of diagnostic investigations and management strategies. Four urologists and one oncologist working in the PROCESS study centres were asked to independently consider 126 hypothetical patients, constructed as combinations of age (<65, 65–74, and 75+ years), PSA (<20, 20–99, 100+ ng ml^−1^), disease stage (localised, locally advanced and metastatic disease), Gleason score (<5, 5–7 and 8+) and comorbidity (low and moderate/high). Each hypothetical patient was rated as being appropriate for a bone scan, a CT scan, radical prostatectomy, radical radiotherapy, hormones only and watchful waiting on a 1–9 scale, with 1–3 being inappropriate, 4–6 being equivocal and 7–9 being appropriate. For each hypothetical patient, a median of the five ratings was taken for the appropriateness of each procedure. Each PROCESS cohort member was then matched to a hypothetical patient according to the abovementioned categories of age, PSA, stage, grade and comorbidity, and was thus matched to median appropriateness ratings for different investigations and approaches.

The PROCESS study observed that black men are diagnosed 5 years earlier than white men. It concluded that black men lived in less affluent areas and were more likely to be in manual occupation. Knowledge of prostate cancer was comparable, with white men more likely to be aware of age as a risk factor and black men more likely to know of their own higher risk of developing prostate cancer. Black and white men were equally likely to present with comorbid conditions and, although there was weak evidence that black men were less likely to present in the absence of symptoms, this association was attenuated after age adjustment. General practitioners were by far the most common source of referral for all men, but black men were more likely than white men to be referred by the emergency department or another hospital team. There was also no convincing evidence of black men delaying their presentation more than white men, with more than half of all men seeking attention within 3 months of symptoms development. The final conclusion was that there was no evidence that black men had better or worse access to diagnostic services, and higher prostate cancer incidence rate among black men in the UK is genuine (Metcalfe *et al*, 2008).

Diagnostic investigations, clinical stage of disease at presentation and initial management of prostate cancer in black and white men in the UK were analysed and published by the PROCESS study group in 2009. The PROCESS study showed that, although age-adjusted PSA level among black men in the UK were higher at presentation, both black and white men had very similar clinical stage and Gleason scores with three-quarters of both groups being diagnosed while their disease was still localised and Gleason score ⩽7. Black men were more likely to undergo bone, CT and MRI scans, and age-adjusted analysis suggested that this was because of the fact that they were diagnosed at a younger age (Evans *et al*, 2010).

Preliminary data suggest no mortality difference between black and white men in the UK (personal communication with PROCESS authors).

There is a wealth of data from United States showing poorer prognosis among black Americans suffering from prostate cancer. Considering the fact that in America, black men occupy less privileged socioeconomic positions and access to health services is largely determined by the patients’ ability to pay, the poorer prognosis observed in the US black men is not necessarily due to a more aggressive disease type (Shapiro and Oliver, 1996; Evans *et al*, 2008).

As it is difficult to interpret variations in management between black and white men diagnosed with prostate cancer, a ‘Delphi’ consensus approach was used by a panel of experts to ascertain whether management was appropriate for a large number of clinical scenarios in each group. This study demonstrated that black men were more likely than white men to undergo curative treatments for their localised cancer, but age-adjusted analysis indicated that this was because of the younger age of black men at diagnosis (Evans *et al*, 2010).

### Aetiology

#### Genetics

The higher risk of prostate cancer among family members and certain ethnicities, such as African Americans, prompted the hypothesis that genetic factors partly account for this difference. Prostate cancer has been the most productive cancer in terms of susceptibility loci identified through genome-wide association studies, with at least 15 loci identified to date. The first and most important region to emerge was 8q24 (8 is the chromosome number, q means the long arm of the chromosome and 24 is the position on the long arm; Amundadottir *et al*, 2006).

Subsequent analyses of genome-wide association studies data by the deCode group, based on a scan of 1500 men with prostate cancer, identified two further loci on 17q (Gudmundsson *et al*, 2007).

At least three distinct loci in separate linkage disequilibrium blocks are present on 8q24, all of which have been confirmed in subsequent genome-wide association studies (Gudmundsson *et al*, 2007; Yeager *et al*, 2007; Eeles *et al*, 2008; Thomas *et al*, 2008).

Analyses by Haiman *et al* identified at least seven or more independent risk alleles in these blocks (Haiman *et al*, 2007).

Intriguingly, the susceptibility alleles at all these loci are more common in Africans and African Americans, and thus explain, at least in part, the higher frequency of the disease in men with African ancestry (Freedman *et al*, 2006).

In the largest study until now, Eeles *et al* (2008) conducted a genome-wide association studies using 2000 diagnosed prostate cancer cases below the age of 60 years or with a family history of the disease identified seven novel loci on chromosomes 3, 6, 7, 10, 11, 19 and X .

#### Hormones

Although the study by Hsing and Comstock, 1993, compared patients with prostate cancer with people in control groups, found no difference in levels of testosterone and dehydrotestosterone, other studies have found that young black men have testosterone levels that are 15% higher than that in young white men (Ross *et al*, 1986; Wu *et al*, 1995).

Furthermore, evidence indicates that 5-alpha reductase may be more active in black men than in white men, implying that hormonal differences may have a role in increased risk of prostate cancer among black populations (Ross *et al*, 1992).

#### Diet

A high-fat diet may lead to increased risks, whereas a diet rich in soy may be protective. These observations have been proposed as reasons for the low prevalence of this cancer in Asia. Rates of prostate cancer are much greater in Japanese-American men than in native Japanese men, supporting the association of a high-fat diet with cancer. Perhaps the same can be applied to black men living in developed countries. Cell culture studies have shown that omega-6 fatty acids are positive stimulants of prostate cancer cell growth, whereas omega-3 fatty acids are negative stimuli. These fats may exert their effects by alterations of sex hormones or growth factors or through effects on 5-alpha reductase (Committee on Diet, Nutrition and Cancer, 1982; Maclean *et al*, 2006).

It has been postulated that vitamins D and soy may have a protective effect against prostate cancer, whereas there is a controversy around the protective effect of vitamin E and selenium. The Selenium and Vitamin E Cancer Prevention Trial concluded that vitamin E and selenium alone or in combination did not prevent prostate cancer in a population of 35 533 relatively healthy men ( Lippman *et al*, 2009).

Whether there is any significant difference in the level of those substances in the diets of black and white men is yet to be investigated (Kolonel *et al*, 1999; Moyad, 1999; Klein *et al*, 2003; Bjelakovic *et al*, 2007).

## Discussion

On the basis of available data, it is clear that black men have higher risk for developing prostate cancer than white men. However, the possible ethological factors and whether prostate cancer has a different biology in black men compared with white men are yet to be determined. The published difference in prostate cancer incidence rate between white and black men implies that there are genetic factor(s) involved, making black men more susceptible to prostate cancer. Although a number of possible genetic mutations has been identified, this area needs further exploration. The difference in incidence rate among black men in various countries suggests that environmental factors such as diet and socioeconomic status influence the incidence rate. Having higher incidence rate among black men in Western countries could either mean that the detection rate is higher in developed countries, or that western lifestyle increases the incidence or both. The younger age of presentation and higher PSA observed in black men with prostate cancer in the UK study (PROCESS) needs to be taken into consideration should there be any screening programme in future for prostate cancer in the UK or Europe.

By comparing the significant different presenting features and outcomes seen between black and white Americans, with more equal outcomes observed between white and black men in the UK, it could be concluded that the behaviour or biology of prostate cancer is similar in different ethnicities and equal access to urological services can result in equal outcomes.

It was shown by The European Randomized Study of Screening for Prostate Cancer that 1410 men would need to be screened and 48 additional cases of prostate cancer would need to be treated to prevent one death from prostate cancer. The study concluded that PSA-based screening reduced the rate of death from prostate cancer by 20%, but was associated with a high risk of overdiagnosis (Schroder *et al*, 2009).

Therefore, the question of the relevance of mass screening remains unresolved. The financial impact, the risk of overdiagnosis and overtreatment are the main obstacles to its implementation. However, it would be logical to consider that perhaps in certain ethnicities with higher incidence rates of prostate cancer, such as African-Caribbean men, a targeted screening may be justified.

Raising the awareness of prostate cancer in African-Caribbean community and implementing the National Awareness and Early Diagnosis Initiative, led by Department of Health and Cancer Research UK will provide a vehicle for earlier diagnosis and better outcome particularly for those at higher risk of prostate cancer.
